# Plasma proteomics reveals the molecular features in rapidly progressive pneumoconiosis

**DOI:** 10.3389/fimmu.2026.1833502

**Published:** 2026-08-14

**Authors:** Meian Tang, Yufei Luo, Ying Li, Weiwei Tang, Xia Yi, Xiaohua Zhang, Liming Wang, Rou Chen

**Affiliations:** 1 The First Affiliated Hospital of Changsha Medical University, Changsha, China; 2 School of Pharmacy, Hunan Vocational College of Science and Technology, Changsha, China; 3 Hunan Prevention and Treatment Institute for Occupational Diseases, Affiliated Prevention and Treatment Institute for Occupational Diseases of University of South China, Hunan Province Clinical Medical Research Center for Occupational Diseases, Changsha, China; 4 School of Biomedical Sciences, Hunan University, Changsha, China; 5 School of Nursing, Hunan University of Chinese Medicine, Changsha, China

**Keywords:** rapidly progressive pneumoconiosis, proteomic profiling, pulmonary fibrosis, lung function, macrophage

## Abstract

**Background:**

Rapidly progressive pneumoconiosis (RPP) is an aggressive subtype of pneumoconiosis characterized by persistent pulmonary inflammation, rapidly progressive fibrosis, and early-onset progressive massive fibrosis. However, the systemic molecular alterations of RPP remain unclear. Here, we profiled the plasma proteome to identify candidate proteins associated with RPP, aiming to discover potential peripheral biomarkers for this aggressive phenotype.

**Methods:**

Plasma samples from 15 RPP patients and 13 non-RPP controls were analyzed by data-independent acquisition (DIA)-based proteomics. Differential expression analysis, protein–protein interaction network analysis, and random forest machine learning were performed for identifying RPP-associated proteins. Subsequently, the least absolute shrinkage and selection operator (LASSO) regression was applied to prioritize candidate biomarkers, whose associations with lung function were then evaluated using correlation analysis. Finally, the expression levels of these key proteins were validated in an independent cohort and a cell model using western blot and enzyme-linked immunosorbent assay.

**Results:**

A total of 74 differentially expressed proteins (DEPs) were identified in RPP compared to non-RPP. These DEPs were predominantly enriched in pathways involving macrophage activation, glycolysis/gluconeogenesis, complement and coagulation cascades, cytoskeletal reorganization, and neutrophil extracellular traps. Through DEPs-based screening, six proteins were identified as key molecules of RPP. Five of them showed significant correlations with lung function. The protein expressions of F11, CSTB, PSMB9, PRB4, and DLAT were validated in an independent cohort and crystalline silica-induced cell model.

**Conclusions:**

This study provided a systematic plasma proteomic profiling of RPP, revealed coordinated alterations in immune, metabolic, and coagulation pathways, and identified a prioritized panel of candidate biomarkers for future validation. These findings constituted a foundational resource for elucidating the molecular architecture of RPP.

## 1 Introduction

Pneumoconiosis, an irreversible and chronic pulmonary disease, typically progresses over decades, even following the cessation of further exposure to silica (1, 2). However, recent studies have described a severe form of pneumoconiosis, named rapidly progressive pneumoconiosis (RPP). Unlike ordinary pneumoconiosis, RPP is characterized by rapid radiological progression, young age at onset, short latency, high mortality and poor prognosis (3–5). The high prevalence of RPP among patients with pneumoconiosis is evidenced by epidemiological data, such as a survey on coal workers’ pneumoconiosis which reported a rate of 35.4% (5). Our previous study demonstrated a rapid increase size of progressive massive fibrosis (PMF), with a median increase of 21.9% after 15 months of follow-up in pneumoconiosis (6). The situation has been exacerbated by the widespread use of artificial stone, with 56% of patients showing rapid disease progressed over a mean follow-up of 4.01 ± 2.1 years (7). Currently, the diagnosis of RPP mainly relies on longitudinal radiographic follow-up (3, 5). Most RPP patients already present with severe pulmonary fibrosis or established PMF and marked loss of lung function at the time of diagnosis, which inevitably leads to premature death (4, 7, 8). Therefore, there is an urgent need to explore reliable biomarkers for RPP for diagnosis and intervention.

Blood, as the most readily accessible sample in clinical diagnosis, contains many biological molecules, which can indicate the occurrence and progression of diseases (9, 10). Plasma proteins are the most high abundance of functional component in human blood and play a dominant role in various biological processes such as signal transduction, transport and anti-infection (11). Mass spectrometry-based proteomics can quantify thousands of plasma proteins and provide unique insights for the dysregulated pathways and molecules, which are widely used to exploring the pathological processes in diseases. Recently, plasma proteomic coupled with bioinformatics technology has been widely employed to investigate the pathogenesis of various lung diseases. *Wang* et al. identified a biomarker panel associated with the pathophysiology of idiopathic pulmonary fibrosis by serum proteomic profile (12). *Bar J* et al. revealed potential biomarkers for immune checkpoint inhibitors-based therapeutic benefit by analyzing the plasma proteomic profiles of non-small cell lung cancer patients (13). *Huang Y* et al. developed a blood-based protein classifier by high-throughput proteomics assays, which can distinguish connective tissue disease-associated interstitial lung disease from idiopathic pulmonary fibrosis (14). However, to date, no study has utilized plasma proteomic to investigate the relationship between plasma proteins and RPP.

In this study, we employed data-independent acquisition (DIA)-based proteomics to profile the plasma proteomic landscape of RPP, identifying key proteins associated with disease development. These results expand the current understanding of RPP-associated molecular alterations at the systemic level and nominate candidate biomarkers for future investigation.

## 2 Materials and methods

### 2.1 Study population and data sources

**Figure 1:**
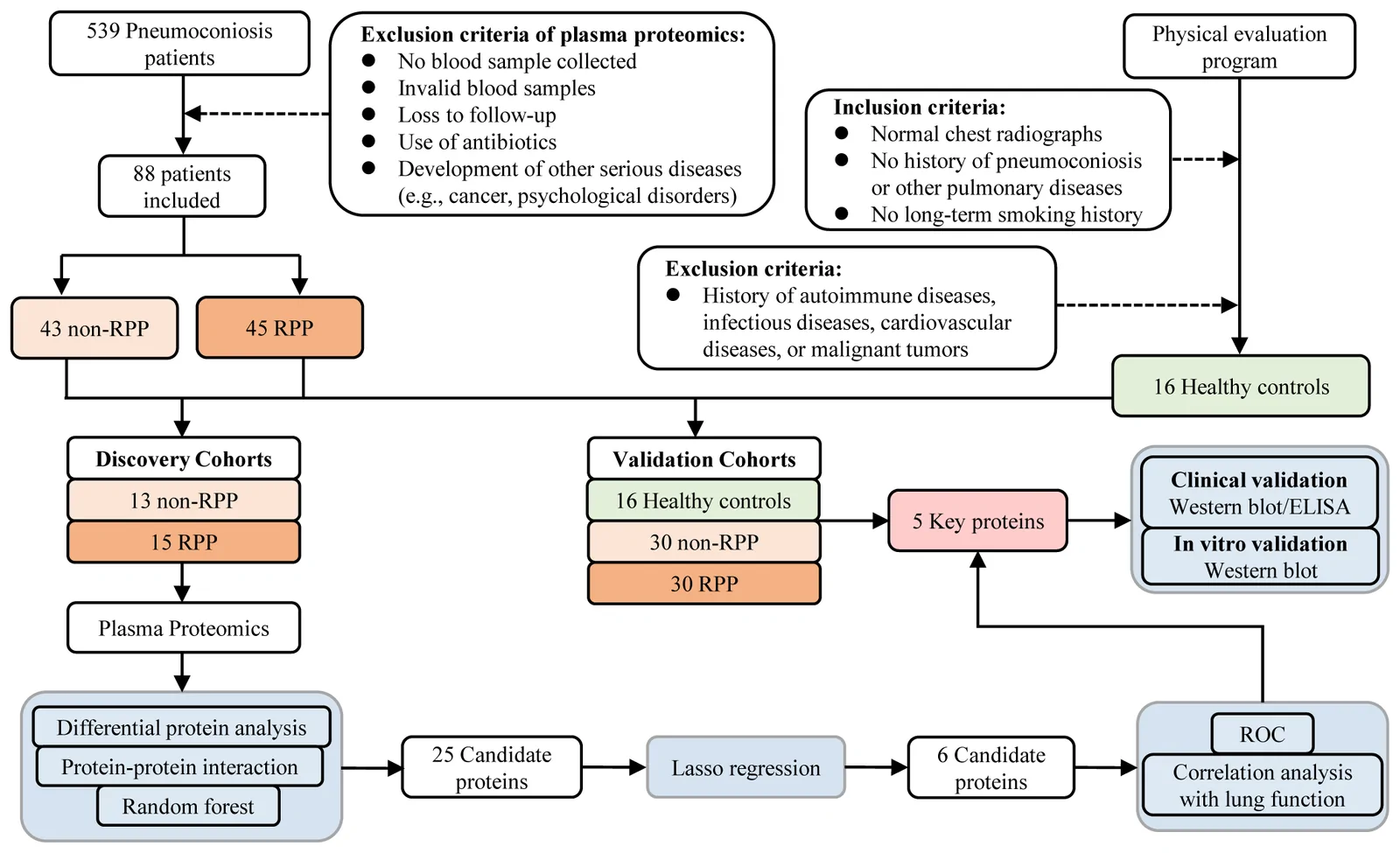
The workflow for molecular features screening.

We recruited patients with pneumoconiosis in Hunan Prevention and Treatment Institute for Occupational Diseases from January 2017 to June 2020. All pneumoconiosis patients were followed up regularly in hospital consultations with annual chest radiograph and respiratory function tests. Then, two specialized radiologists classified pneumoconiosis patients into RPP or non-RPP according to the radiologic criteria. RPP was defined according to two complementary criteria: (a) an increase of more than one International Labor Organization (ILO) subcategory in small opacity profusion within five years (Class 1, reflecting radiologic progression), or (b) the presence of PMF in a miner younger than 70 years or with a dust exposure tenure shorter than 40 years (Class 2, reflecting early-onset or short-latency severe disease) (4). Correspondingly, patients were classified as non-RPP who were not diagnosed with RPP over a follow-up duration of approximately five years or longer. Plasma samples were subsequently collected from 45 RPP and 43 non-RPP patients upon confirmation of their respective diagnoses (**Supplementary Table 1**). Patients were excluded if they had one of the following conditions: development of other serious diseases (e.g., cancer, psychological disorders); use of antibiotics; loss to follow-up; unqualified blood samples; uncollected blood sample. Then, we selected 28 patients (15 RPP and 13 non-RPP) with significant radiograph differences between two groups to explore the plasma proteomics (**Supplementary Tables 1**, **2**). The remaining patients served as the validation cohort. Detailed inclusion and exclusion criteria for all patients can be found in **Figure 1**. In addition, 16 healthy controls were recruited from the Health Management Center of Hunan Prevention and Treatment Institute for Occupational Diseases. The inclusion criteria for healthy participants were normal chest radiographs, no history of pneumoconiosis or other pulmonary diseases (including asthma, chronic obstructive pulmonary disease, tuberculosis, or interstitial lung disease), and no long-term smoking history (defined as > 10 pack-years). Exclusion criteria included the presence at enrollment of autoimmune diseases, infectious diseases, cardiovascular diseases, or malignant tumors. The study was approved by the Hunan Prevention and Treatment Institute for Occupational Diseases Ethics Committee (Approval number: LS2021122401), and informed consent was obtained from all participants.

### 2.2 DIA proteomics

Morning fasting venous blood samples (2 mL per patient) were collected from the cephalic vein of 28 patients. Then, blood samples were allowed to stand at room temperature for 3 h and centrifuged for 7 min (3000 rpm) to obtain plasma. The top 14 high abundance proteins were removed by Pierce™ Top 14 Abundant Protein Depletion Spin Columns Kit (ThermoFisher Scientific), according to the manufacturer’s instructions. Following depletion, the proteins were denatured, reduced, alkylated, digested into peptides, and desalted using a C-18 column. The tryptic peptides were dissolved in solvent A, directly loaded onto a home-made reversed-phase analytical column (25-cm length, 100 μm i.d.). The mobile phase consisted of solvent A (0.1% formic acid, 2% acetonitrile/in water) and solvent B (0.1% formic acid, 90% acetonitrile/in water). Peptides were separated with the following gradient: 0–16 min, 7%-20%B; 16–24 min, 20%-32%B; 24–27 min, 32%-80%B; 27–30 min, 80%B, and all at a constant flow rate of 500 nl/min on an EASY-nLC 1200 UPLC system (ThermoFisher Scientific). The separated peptides were analyzed in Orbitrap Exploris 480 with a nano-electrospray ion source. The electrospray voltage applied was 2300 V. FAIMS compensate voltage was set as-45 V, -70 V. Precursors and fragments were analyzed at the Orbitrap detector. The full MS scan resolution was set to 30000 for a scan range of 390–810 m/z. The MS/MS scan was fixed first mass as 200 m/z at a resolution of 30000. The HCD fragmentation was performed at a normalized collision energy of 25%, 30%, 35%. Automatic gain control target was set at 3E6, with a maximum injection time of Auto. The data obtained from the mass spectrometry analysis, after the database search is completed, requires a series of quality control evaluations to ensure that the results meet the standards. Then, the data were processed using Spectronaut (v.18) software. Tandem mass spectra were searched against the Homo_sapiens_9606_SP_20220107.fasta (20376 entries) concatenated with reverse decoy database. Trypsin/P was specified as cleavage enzyme allowing up to 2 missing cleavages. Carbamidomethyl on Cys was specified as fixed modification. Acetylation on protein N-terminal, and oxidation on Met were specified as variable modifications. False discovery rate (FDR) of protein, peptide and PSM was adjusted to < 1%. For the missing values, they were simply left as missing and no filling treatment was carried out. Finally, a library containing 9,850 peptides and 1,329 proteins was built. The mass spectrometry proteomic data have been deposited to the ProteomeXchange Consortium (https://proteomecentral.proteomexchange.org) via the iProX partner repository (15, 16) with the dataset identifier PXD063647.

### 2.3 Differential protein and pathway enrichment analysis

For differentially expression analysis, 1,319 proteins with detectable intensity in least two samples in every group were included. Differentially expressed proteins (DEPs) were initially analyzed using Student’s t-tests with Benjamini-Hochberg FDR correction. This approach yielded only four proteins with FDR<0.05 (**Supplementary Table 3**), which is insufficient for downstream biomarker screening. Given the exploratory nature of this study and the known risk of inflated false negatives when applying stringent FDR correction to small-sample proteomic data (17, 18), we adopted a dual-threshold filter (*p* < 0.05 and fold changes >1.5 or <2/3) as the initial screening criterion, consistent with established proteomic biomarker studies (12, 19, 20). Principal component analysis (PCA) plot with DEPs were performed by R software and the missing values were dealt with R package *missMDA*. The R package *ggplot2* (v3.3.3) was used for the volcano plot for DEPs. The immune-related DEPs were obtained by intersecting DEPs with the lists of immune-related genes obtained from ImmPort (http://www.innatedb.com) and InnateDB (https://www.immport.org/home).

The biological characteristics of the DEPs were determined by pathway enrichment analysis with Gene Ontology (GO) and Kyoto Encyclopedia of Genes and Genomes (KEGG, http://www.genome.jp/kegg/). The GO analysis was further divided into biological process (BP), cellular component (CC) and molecular function (MF). The R package clusterProfiler (v4.8.1) (21) was used for the enrichment analysis with human genome (org.Hs.eg.db). The statistical significance of pathway enrichment was determined by Fisher exact test and pathways with FDR<0.05 were regarded as being significantly regulated. The top ten GO terms with BP, CC, MF were showed by R package ggplot2.

### 2.4 Protein-protein interaction network analysis

A PPI network of DEPs was built by using the STRING database (https://string-db.org/). Only interactions between the proteins belonging to the searched data set were selected, thereby excluding external candidates. Proteins with a minimum required interaction score ≥0.4 were chosen to build a full network model, which was then visualized in Cytoscape software (https://cytoscape.org). We used the Degree algorithm of the CytoHubba plugin to select the hub proteins in the network (22). The modules in the PPI network were analyzed using the MCODE plug-in in Cytoscape software with the default parameters (23).

### 2.5 Machine learning–based biomarker selection

The random forest algorithm was employed to screen important protein variables that could distinguish RPP from non-RPP, with a filter condition of relative importance greater than 0.5. Variable importance for each protein was determined by a minimum out-of-bag error rate.

To identify key proteins, we integrated candidate proteins derived from the top DEPs, PPI network analysis, and random forest analysis. The least absolute shrinkage and selection operator (LASSO) regression was then applied using R package *glmnet*. Given the limited sample size (n = 28) relative to the number of candidate proteins (n = 25), we assess the stability of LASSO-based feature selection via a bootstrap resampling procedure with 100 iterations (24, 25). In each iteration, a bootstrap sample (with replacement, same size as the original dataset) was generated. Ten-fold cross-validation (CV) was used to select the optimal penalty parameter lambda (λ) (26). Proteins with non-zero coefficients in each iteration were recorded. The selection frequency (percentage of bootstrap iterations in which a protein was retained a non-zero coefficient) was calculated for each of the 25 candidate proteins. Proteins with a selection frequency ≥ 0.5 were defined as stable key biomarkers. This approach mitigates the risk of overfitting and enhances the robustness of feature selection in small-sample settings. Then, the effectiveness of key proteins was evaluated in the discovery cohort by plotting the receiver operating characteristic (ROC) curve and calculating the area under the curve (AUC) (R package *pROC*) (27). The AUC value is closer to 1, which indicates the better performance of proteins. Diagnostic model cut-off values were determined using the “OptimalCutpoints” package (version 1.1–5) via the Youden method, which maximizes the sum of sensitivity and specificity minus 1. It’s worth noting that the LASSO analysis was employed strictly as an exploratory feature-screening tool to prioritize candidate proteins for subsequent validation; the AUC estimates reported herein should be interpreted with caution and are not intended to represent validated diagnostic performance.

### 2.6 Cell culture

The mouse alveolar macrophage cell line MH-S was procured from Procell (CL-0597, Wuhan, China). MH-S cells were maintained in RPMI 1640 medium enriched with 10% fetal bovine serum (FBS; CellMax, Beijing, China), 0.05 mM β-mercaptoethanol, and 1% penicillin-streptomycin (P/S; GA3502, GENVIEW, Tallahassee, FL, USA). The murine fibroblast NIH-3T3 cell line was obtained from the Stem Cell Bank of the Chinese Academy of Sciences (Shanghai, China). NIH-3T3 cells were cultured in Dulbecco’s Modified Eagle’s Medium supplemented with 10% FBS (CellMax, Beijing, China) and 1% P/S. All cells were incubated at 37 °C in a humidified atmosphere containing 5% CO_2_ and were routinely screened to ensure the absence of mycoplasma contamination. Crystalline silica (CS) was obtained from Didu Pharmaceutical and Chemical Co., Ltd. (Shaanxi, China). To eliminate endotoxin contamination, the particulates were heated at 200 °C for 2 hours and subsequently suspended in PBS. Prior to experimental use, the suspensions were subjected to sonication for 10 minutes to ensure homogeneity.

To model the elevated inflammatory state characteristic of RPP, MH-S macrophages were exposed to CS at graded concentrations for 24 hours. A low dose of CS (25 μg/cm²) was applied to simulate non-RPP conditions, while a high dose (100 μg/cm²) was used to recapitulate RPP-associated injury. Cell viability and proliferation were assessed using the CCK-8 and MTT assays, respectively, and apoptosis was evaluated via propidium iodide (PI) uptake assay, as previously described in our study (28). Conditioned medium (CM) was collected from MH-S macrophages treated with control, low-dose CS, or high-dose CS, and then applied to NIH-3T3 fibroblasts to assess paracrine effects. For wound healing assays, confluent NIH-3T3 monolayers were scratch-wounded and incubated with the respective CM for 24 hours; wound closure was quantified using ImageJ software. In parallel, fibroblast proliferation was evaluated using the CCK-8 assay.

### 2.7 Rationale for CS dose selection in the *in vitro* cell model

RPP represents a severe form of silicosis characterized by massive lung inflammation, rapid progression to pulmonary fibrosis, and substantial lung function decline within a short period, driven by high-concentration exposure to respirable crystalline silica (3, 4). Clinically and pathologically, RPP is distinguished from classic silicosis by the presence of intense and persistent pulmonary inflammation, accelerated fibrotic transformation, and a high prevalence of coalescent nodular lesions (3, 29). Importantly, mineralogical analyses of lung tissues from contemporary RPP patients have demonstrated a significantly higher burden of crystalline silica particles compared to historical controls (e.g., 26.1% vs. 17.8% of total particles, and 47.3×10^8^ vs. 25.8×10^8^ particles/cm³ tissue) (29), confirming that excessive CS exposure is a primary driver of RPP pathogenesis.

To model this accelerated disease *in vitro*, we drew on established *in vivo* and *in vitro* evidence. Previous animal models of accelerated silicosis have successfully replicated RPP-like pathology—including severe pulmonary inflammation, rapid collagen deposition, and early lung dysfunction—by employing high doses of CS (30, 31). In silicosis research, a CS concentration of 40–50 μg/cm² is commonly used to stimulate murine alveolar macrophage cell MH-S to induce inflammatory and fibrotic responses (32, 33). Furthermore, *in vitro* studies have demonstrated that even low doses of CS (5 μg/cm²) can induce rapid DNA damage responses in bronchial epithelial cells within minutes of exposure (34), indicating that CS is highly bioactive at concentrations well below those used in traditional silicosis models.

Based on the clinical hallmark of RPP, namely massive inflammation and rapid fibrosis driven by elevated CS exposure (29), we selected two different CS concentrations for *in vitro* experiments. This design was also consistent with findings from established animal models (30, 31) and commonly used cell culture conditions (32, 33). Specifically, 25 μg/cm² CS was selected as the “non-RPP-like condition” to represent a moderate and predominantly inflammatory state. In contrast, 100 μg/cm² CS was used as the “RPP-like condition” to mimic the high-dose, strongly pro-inflammatory, and pro-fibrotic microenvironment characteristic of RPP. These doses were selected to induce different degrees of inflammation, cell injury, and fibrotic responses, ranging from relatively mild changes to more severe pathological alterations, thereby enabling *in vitro* investigation of RPP-related mechanisms.

### 2.8 Western blot analysis

Total protein from cultured cells was lysed in buffer supplemented with 1% protease and phosphatase inhibitors (Targetmol, USA; Cat# C0001, C0004). High-abundance proteins in the plasma were removed from plasma samples using a Pierce™ Top 14 Abundant Protein Depletion Spin Columns Kit (ThermoFisher Scientific). Protein concentrations were determined using a BCA assay kit (P0009, Beyotime). Equal amounts of protein (30-100 μg) were separated by 8–15% SDS-PAGE and transferred onto PVDF membranes. Membranes were stained with Ponceau S to confirm equal loading (35), blocked with 5% BSA in TBST for 1 h at room temperature, and incubated overnight at 4 °C with primary antibodies. After washing, membranes were incubated with HRP-conjugated secondary antibodies for 1 h at room temperature. In all WB analyses of cell lysates in this study, membranes were stripped with stripping buffer (Abiowell, AWB0167b) after target protein detection and then re-probed with internal control antibodies, consistent with our previously established protocol (36). Protein bands were visualized using UltraSignal ECL reagent (4A Biotech) and quantified with ImageJ software. Detailed antibody information is provided in **Supplementary Table 4**.

### 2.9 Enzyme-linked immunosorbent assay

Human serum samples and supernatants from MH-S cells were collected. The concentrations of Human Cystatin-B (CSB-E16835h) and Human Coagulation Factor XI (CSB-EL007916HU) were quantified using ELISA kits from Cusabio, following the manufacturer’s protocols. Additionally, levels of IL-1β (88-7013-88), TNF-α (88-7324-88), and IL-6 (88-7064-88) were measured using ELISA kits (Invitrogen, Carlsbad, CA, United States) as previously described in our study (37).

### 2.10 Statistical analysis

Continuous variables were expressed as the mean ± SD or median (interquartile range) and were analyzed by the Student’s t-test or Mann–Whitney U test. Categorical variables were described as the quantity (percentage) and were compared by the Fisher’s exact test. To assess the rate of decline in lung function parameters over time, a fixed effects linear regression model was performed, as patients had dissimilar time points of observations for data collection. Pearson’s correlation coefficient was used to determine the correlation between key proteins and lung function (R package *corrplot*). A *p*-value of <0.05 was considered statistically significant differences. The Benjamini-Hochberg FDR method was applied for multiple testing correction. All data were analyzed using SPSS 25.0 software (IBM, New York, USA) and R software (version 4.3.1).

## 3 Results

### 3.1 Clinical characteristics of participants

A total of 28 pneumoconiosis samples (15 RPP and 13 non-RPP) were used for proteomic analysis. The demographic and clinical characteristics of participants were summarized in **Table 1**. All patients were men, with a mean follow-up of 4.07 ± 1.00 years. No significant differences were observed in age, BMI, exposure time, pneumoconiosis type, and smoking status between the two groups. Compared with non-RPP group, patients with RPP had higher disease stage (*p* = 0.001).

**Table 1:** Demographic and clinical characteristics of participants in two groups.

Characteristics	non-RPP (n=13)	RPP (n=15)	t/*Z* value	*p* value
Age, years	54.3 ± 4.9	55.7 ± 6.2	0.666	0.511
BMI, kg/m^2^	25.0 ± 3.3	23.4 ± 2.7	-1.409	0.171
Exposure duration, years	20.0 (14.3, 23.8)	14.2 (6.0, 23.0)	-0.738	0.461
Types of pneumoconiosis				
Coal workers’ pneumoconiosis	10 (76.9%)	7 (46.7%)	-^#^	0.137
Silicosis	3 (23.1%)	8 (53.3%)		
Stage of pneumoconiosis				
I	7 (53.8%)	3 (20.0%)	–	<0.001
II	6 (46.2%)	2 (13.3%)		
III	0 (0.0%)	10 (66.7%)		
Smoking history				
Smokers	6 (46.2%)	8 (53.3%)	–	0.062
Ex-smokers	3 (23.1%)	7 (46.7%)		
Non-smokers	4 (30.8%)	0 (0.0%)		
Types of work				
Coal mining	9 (69.2%)	4 (26.7%)	–	0.056
Air drill	4 (30.8%)	10 (66.7%)		
Rock breaking	0 (0.0%)	1 (6.7%)		
Complicated with COPD	1 (7.7%)	5 (33.3%)	–	0.173

Smoking history were categorized as non-smokers, smokers and ex-smokers (had quit smoking ≥12 months). ^#^Fisher’s exact test.

The chest radiographs were compared between baseline and last follow-up in these two groups. In non-RPP group, no PMF patients appeared and the small opacity profusion did not increase more than one ILO subcategory even following more 5 years. However, the radiographic progression in RPP group was faster, with the prevalence of PMF increasing from 40% (6/15) to 100% (15/15) and the prevalence of category C increasing from 13.3% (2/13) to 53.3% (8/13) (**Supplementary Table 2**). Additionally, there were significant differences in the size of opacity between baseline and last follow-up in RPP (0.0 cm vs 72.0 cm, *p* < 0.001, **Supplementary Table 2**).

### 3.2 The proteomic profiling of RPP and non-RPP

**Figure 2:**
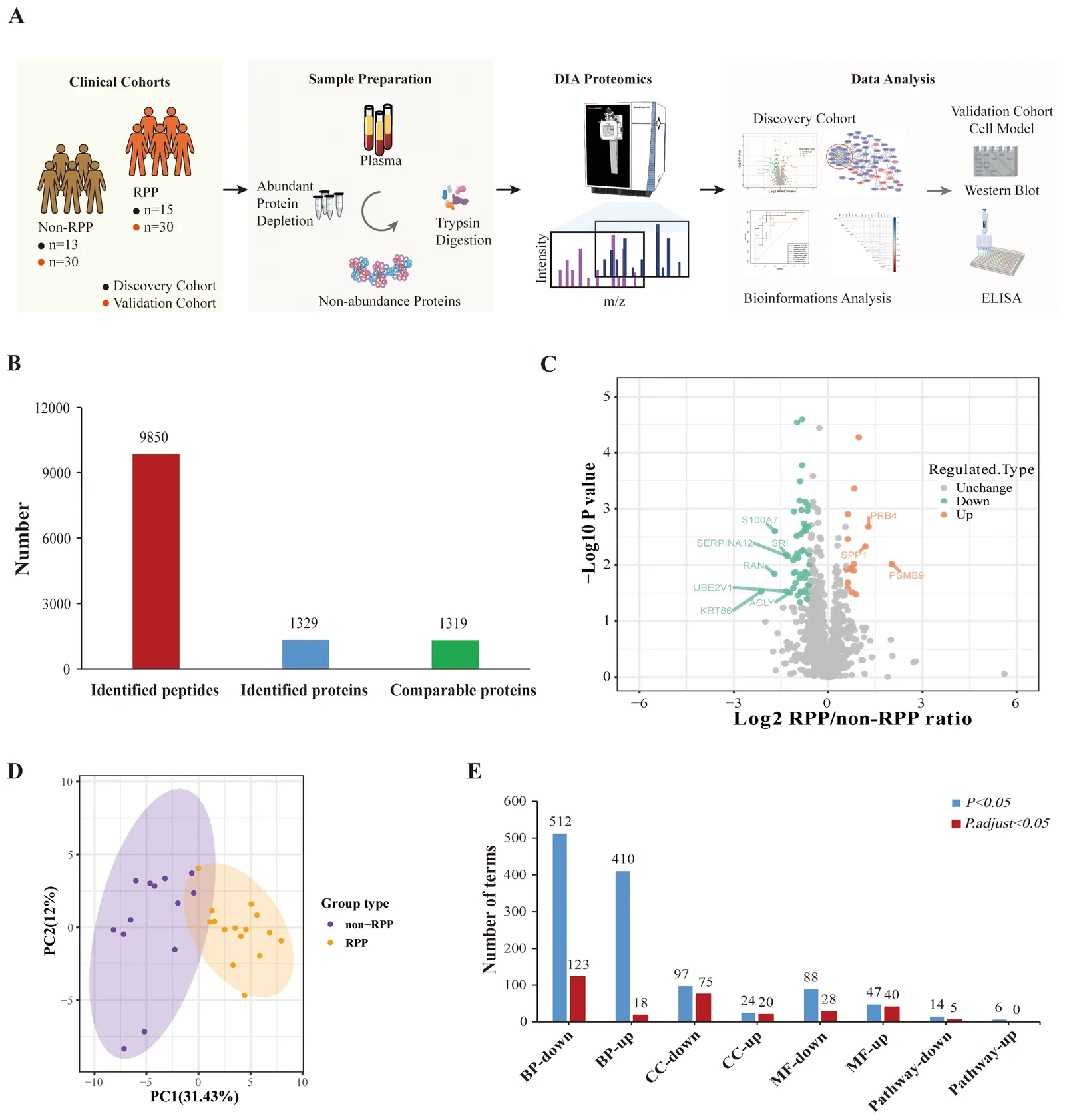
Plasma protein profiles in non-RPP (n=13) vs RPP (n=15) groups. **(A)** Experimental workflow for proteomic analysis. **(B)** Statistics for the peptides and proteins identification and comparable proteins. The comparable proteins refer to proteins which has expression intensity at least two samples in every group. **(C)** Volcano plot with the magnitude expressed as log2 fold change (x-axis) and significance expressed as -log10 of the p value (y-axis) of differential expression analysis. The top 10 differentially expressed proteins were labelled. **(D)** PCA analysis of the proteomic profiling in the RPP group and non-RPP group. **(E)** Statistics for the GO terms including BP, MF, and CC, and KEGG pathway annotation. BP, biological process; MF, molecular function; CC, cellular component.

To gain molecular insights associated with RPP pathogenesis, we used a DIA-based quantitative proteomic workflow to profile proteome alterations between the RPP and non-RPP groups (**Figure 2A**). The mass spectrometry results of quality control showed that most peptide segments were distributed within 7 to 20 amino acids, which was in line with the general pattern based on enzymatic digestion and mass spectrometry fragmentation. The identified proteins were present in different ranges of molecular weight and were evenly distributed (**Supplementary Figures 1A–C**). The distribution of protein intensity values among different samples is consistent (**Supplementary Figure 1D**). These results indicated the stability of our mass spectrometry data. Finally, a total of 9850 peptides, 1329 proteins were identified and 1319 quantifiable proteins were compared and analyzed, respectively (**Figure 2B**). Compared with the non-RPP group, a total of 74 DEPs were identified in RPP group (1.5-fold change cutoff and *p* < 0.05), among which 18 proteins were up-regulated and 56 proteins were down-regulated (**Figure 2C**; **Supplementary Table 3**). PCA plot revealed a clear clustering pattern that distinguished the RPP group from the non-RPP group (**Figure 2D**). Notably, a panel of immune-related DEPs were identified, including S100A8, ADIPOQ, F11, SPP1, ANXA2, ILF3, CTSG, PRDX1, IGHV5-10-1, CSF1, JAG1, LTBP2 and PDGFB (**Supplementary Table 3**).

**Figure 3:**
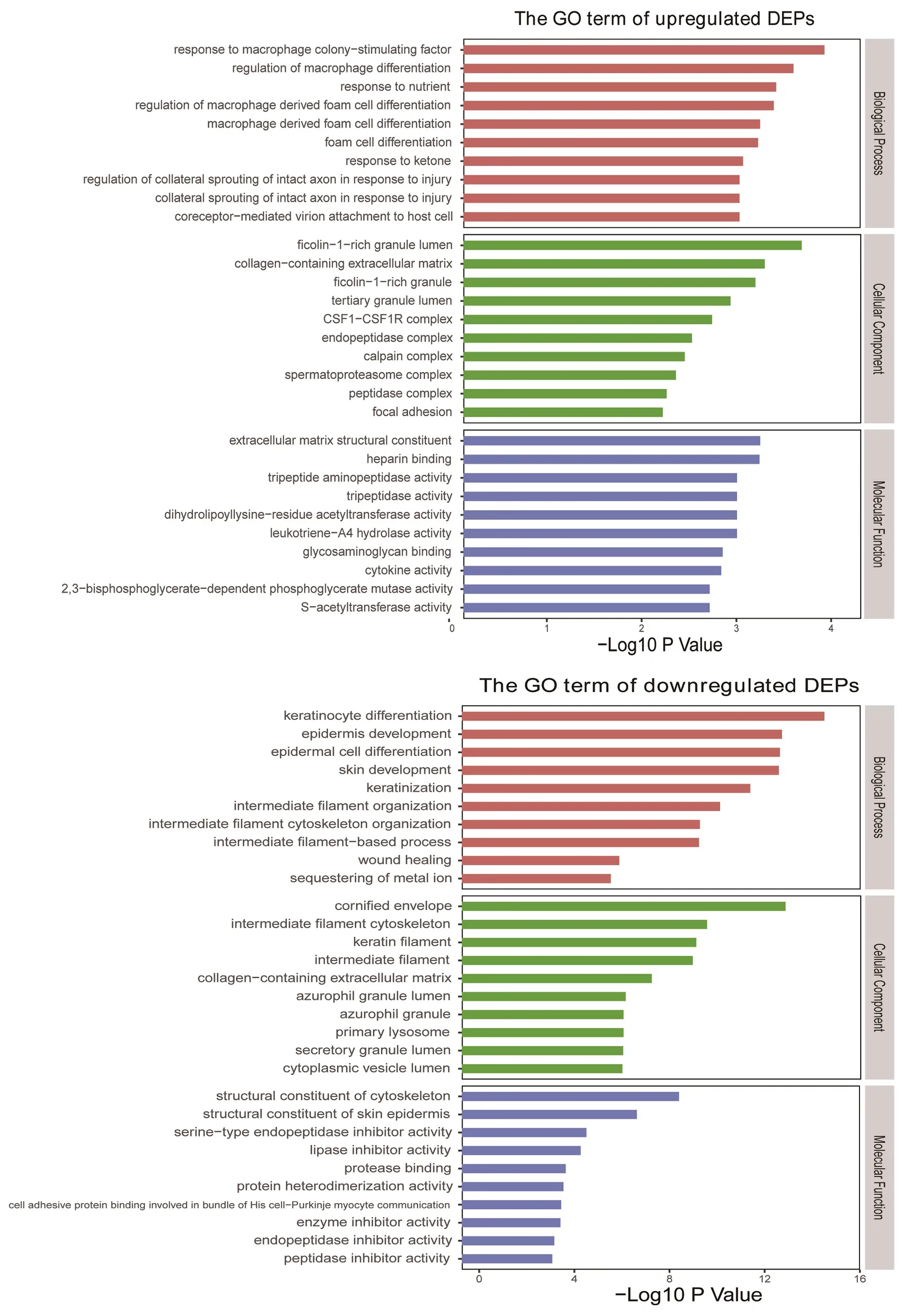
GO analysis of differentially expressed proteins between the RPP and non-RPP groups. The top ten biological process terms, cellular component terms and molecular function terms are presented.

To further explore the biological significance of the DEPs, we performed the GO and KEGG pathways functional annotation. The results showed that a total of 18 BP terms, 20 CC terms, 40 MF terms in up-regulated DEPs and 123 BP terms, 75 CC terms, 28 MF terms and 5 pathways in down-regulated DEPs were identified with *p*. adjust < 0.05, respectively (**Figure 2E**; **Supplementary Table 5**). Taken BP as an example, most of the BP terms in up-regulated DEPs were associated with macrophage cell differentiation, macrophage colony-stimulating factor, nutrient responding and extracellular matrix structural constituent, while the downregulated DEPs were enriched in keratinocyte differentiation, epidermis development, intermediate filament-based process like cytoskeleton organization, and wound healing (**Figure 3**). The KEGG pathways enrichment analysis showed that the up-regulated DEPs were enriched in the glycolysis/gluconeogenesis, carbon and lipoic acid metabolism, focal adhesion, TCA cycle, while the down-regulated DEPs were involved in neutrophil extracellular trap formation, estrogen signaling pathway, complement and coagulation cascades and mitophagy (*p* < 0.05, **Supplementary Table 6**).

### 3.3 Screening the key proteins from DEPs–based analysis

**Figure 4:**
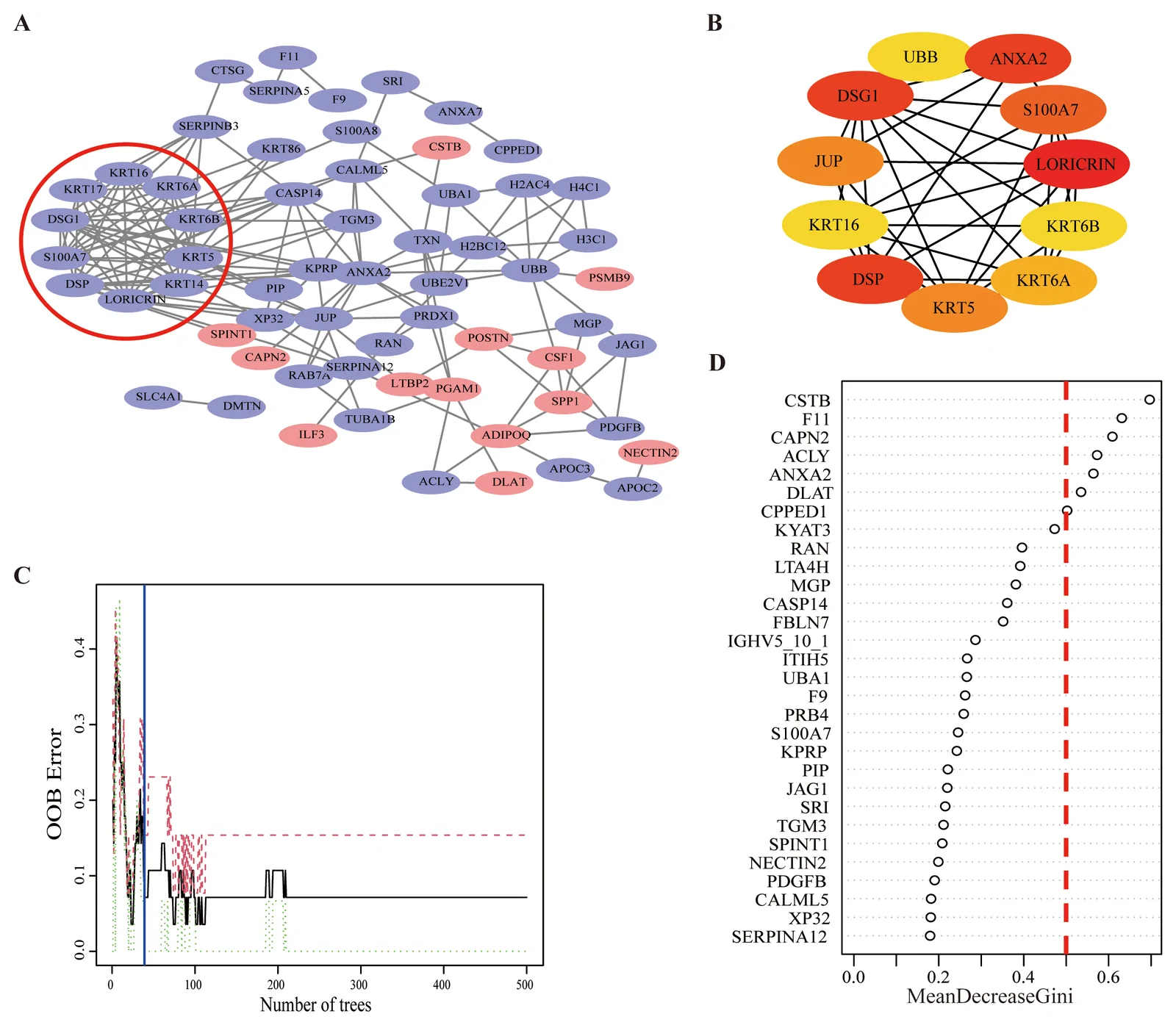
The candidate proteins from PPI–based screening and machine learning analysis. **(A)** PPI network of module analysis. The proteins in red circle constituted the key module. Rose red indicates the up-regulated proteins, whereas purple indicates the down-regulated proteins. **(B)** Hub proteins from the PPI network. The color of node is redder; the degree scores is higher. **(C)** Out of bag error rate reached a minimum when the number of trees was equal to 39. **(D)** Relative importance of top 30 proteins calculated in random forest with the MeanDecreaseGini parameter.

A PPI network representing the relationship of proteins encoded by DEPs was generated using the STRING database. The results were downloaded and analyzed using Cytoscape software. One functional module was screened from the PPI network using MCODE, as shown in **Figure 4A**. The biological functions of proteins in module were related to keratinocyte, epidermis, intermediate filament-based process like cytoskeleton organization (**Supplementary Table 7**). In addition, the 11 hub proteins were screened according to their degree values >10, and these hub proteins were also belonged to module proteins except for UBB (**Figure 4B**; **Supplementary Table 8**). Then, random forest algorithm was employed to screen protein variables capable of distinguishing RPP and non-RPP, with a relative importance greater than 0.5 as the filtering criterion. The out-of-bag error rate reached a minimum when the number of trees was equal to 39 (**Figure 4C**), and 7 of 74 DEPs were selected as important protein variables (**Figure 4D**).

**Figure 5:**
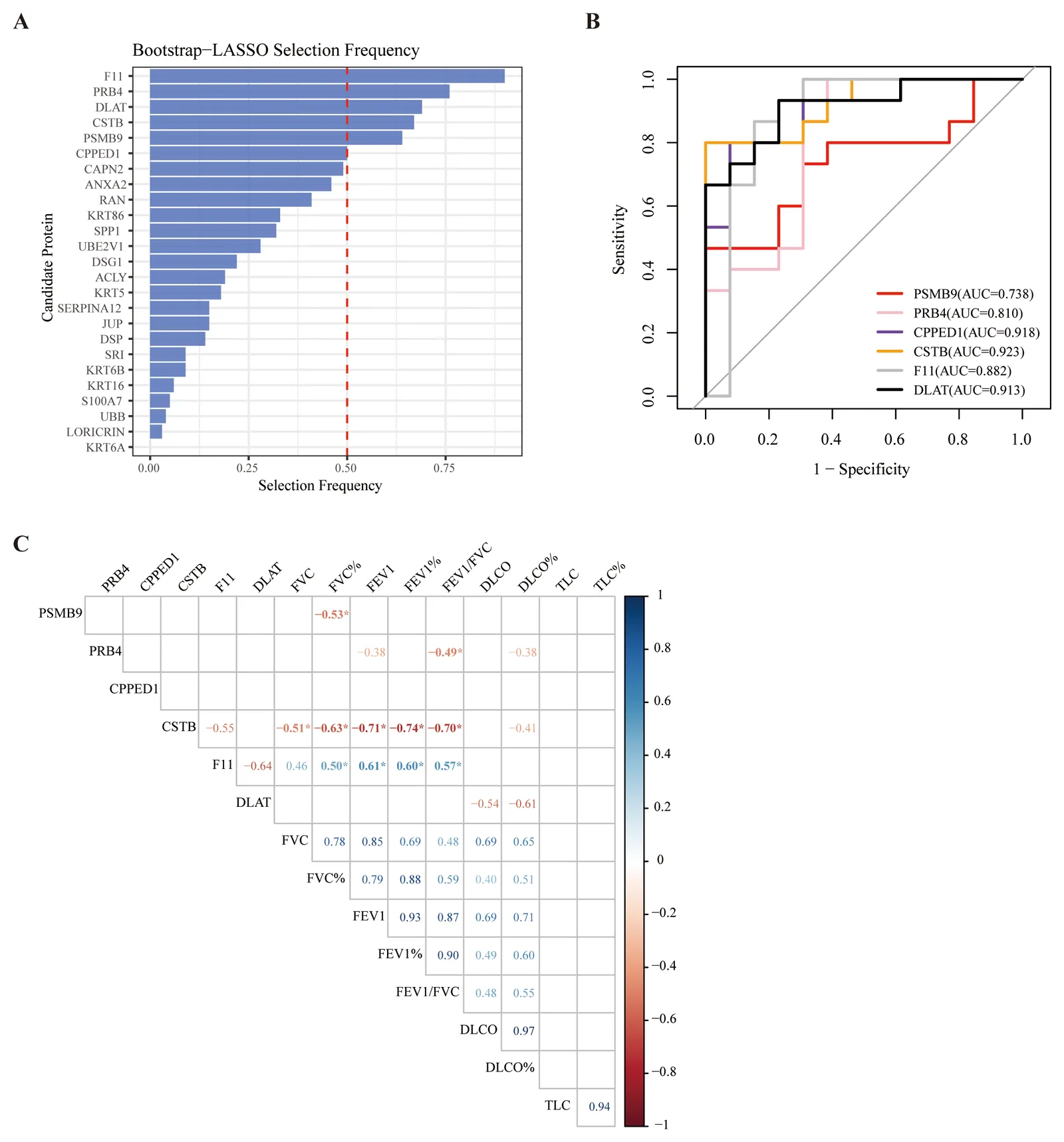
The key proteins from LASSO regresion and correlation analysis. **(A)** Selection frequencies of 25 candidate proteins from 100 bootstrap LASSO iterations. The dashed red line indicates the stability threshold of 0.5. Proteins with frequency ≥ 0.5 (n = 6) were defined as candidate biomarkers. **(B)** ROC analysis of the six key proteins for RPP diagnosis. **(C)** Pearson correlation analysis between key proteins and lung function. For clarity, only correlation coefficients with *p* < 0.05 were shown in the figure. Asterisks indicate correlations that remained statistically significant after FDR correction (*q* < 0.05). The correlation coefficient greater than 0 indicates positive correlation, which means the higher expression of this protein has the stronger lung function, and vice versa.

Given the weak but methodologically complementary correlations among DEPs-enriched GO pathways, PPI hub proteins, and machine learning-selected features, we merged the top DEPs, PPI hub proteins, and machine learning-selected features to leverage their respective strengths and minimize the bias of any single approach. This integrative set yielded 25 candidate proteins which were then subjected to final LASSO regression modeling. To evaluate the robustness of feature selection under the limited sample size (n = 28), we performed a bootstrap resampling stability analysis with 100 iterations. Among the 25 candidate proteins, six had a selection frequency ≥ 0.5 and were therefore defined as stable key biomarkers (λ=0.0002, **Figure 5A**). The AUCs of these six proteins (F11, PRB4, DLAT, CSTB, PSMB9, and CPPED1) for RPP diagnosis were 0.882, 0.810, 0.913, 0.923, 0.738, and 0.918, respectively (**Figure 5B**), suggested their potential utility in discriminating RPP from non-RPP cases in this exploratory discovery cohort.

### 3.4 Analysis of the correlation between key proteins and lung function

Pneumoconiosis is characterized by pulmonary inflammation and diffuse fibrosis caused by inhalation and retention of dust particles in the lung, leading to lung function decline or respiratory failure (38). Our results showed that RPP patients experienced rapid deterioration in lung function than non-RPP patients (**Table 2**). The average annual decline of FEV_1_ was 0.046 L in non-RPP group, whereas it was 0.118 L in RPP group (*p* = 0.014). The average annual decline of FEV_1_/FVC was 0.667% vs 2.109% in non-RPP group and RPP group (*p* = 0.014). The same trend could be seen in FVC and DLCO, with 0.028 L vs 0.073 L and 0.155 mmoL/min/mmHg vs 0.235 mmoL/min/mmHg, respectively. The similar results were also observed in validation cohort (**Supplementary Table 9**).

**Table 2:** Progression of lung function in two groups.

Variations	non-RPP				RPP				*p* value ^b^
	Baseline	Last follow-up	Overall follow-up, annual decrease	*p* value ^a^	Baseline	Last follow-up	Overall follow-up, annual decrease	*p* value ^a^	
FVC, L	3.77 ± 0.40	3.62 ± 0.45	0.028 (0.008,0.048)	0.010	3.56 ± 0.34	3.31 ± 0.46	0.073 (0.011,0.135)	0.025	0.197
FVC% predicted	101.89 ± 8.46	99.18 ± 10.46	0.531 (-0.205,1.266)	0.142	93.28 ± 6.24	88.09 ± 11.12	1.584 (-0.148,3.020)	0.032	0.020
FEV_1_, L	3.00 ± 0.44	2.78 ± 0.46	0.046 (0.031,0.061)	<0.001	2.55 ± 0.37	2.13 ± 0.55	0.118 (0.058,0.177)	0.001	0.014
FEV_1_% predicted	100.96 ± 12.62	93.72 ± 13.04	1.457 (0.766,2.149)	0.001	82.39 ± 8.33	70.41 ± 17.67	3.619 (1.511,5.727)	0.002	0.001
FEV_1_/FVC	79.60 ± 6.65	76.37 ± 5.62	0.667 (0.274,1.060)	0.003	71.69 ± 7.32	63.73 ± 11.91	2.109 (0.794,3.424)	0.004	0.014
DLCO, mmoL/ min/mmHg	7.32 ± 1.65	6.58 ± 2.01	0.155 (0.071,0.238)	0.002	6.49 ± 1.09	5.61 ± 1.49	0.235 (0.065,0.404)	0.010	0.158
DLCO% predicted	85.00 ± 16.94	76.55 ± 20.21	1.756 (0.682,2.831)	0.004	73.96 ± 10.05	63.85 ± 14.56	2.570 (0.412,4.729)	0.023	0.058
TLC, L	6.59 ± 0.90	6.54 ± 0.57	0.009 (-0.060,0.079)	0.782	6.55 ± 0.82	6.88 ± 1.70	-0.048 (-0.257,0.162)	0.645	0.923
TLC% predicted	109.09 ± 14.03	110.10 ± 8.29	-0.197 (-1.493,1.098)	0.746	104.23 ± 11.61	112.31 ± 30.58	-1.427 (-5.089,2.236)	0.434	0.648

Values are expressed as mean ± SD. Lung function data are at baseline and last follow-up. ^a^*p* value for mean reduction for the lung function parameter in the overall period. ^b^*p* value for mean reduction for the lung function parameter when comparing baseline and last follow-up. FVC, forced vital capacity; FEV1, forced expiratory volume in 1 s; DLCO, diffusing capacity of the lung for carbon monoxide; TLC, total lung capacity.

Therefore, we further investigated the association between 6 key proteins and lung function. As shown in **Figure 5C**, F11, PRB4, CSTB, DLAT, and PSMB9 exhibited significant correlations with lung function parameters (*p* < 0.05). After FDR correction, F11, PRB4, CSTB and PSMB9 remained statistically significant (*q* < 0.05). Specifically, the higher plasma CSTB levels were associated with poorer lung function, as evidenced by negative correlations with FVC, FVC%, FEV1, FEV1%, and FEV1/FVC. Similarly, PRB4 and PSMB9 were linked to reduced lung function, showing negative correlations with FVC% and FEV1/FVC, respectively. In contrast, F11 showed positive correlations with FVC%, FEV1, FEV1% and FEV1/FVC, suggesting that lower plasma F11 levels corresponded to worse lung function. The complete correlation matrix, including raw and adjusted p-values, is provided in **Supplementary Table 10**. Additionally, only CSTB was positively associated with the size of opacity (r=0.78, *p* < 0.05).

### 3.5 Validation of lung function-related key proteins in an independent cohort

**Figure 6:**
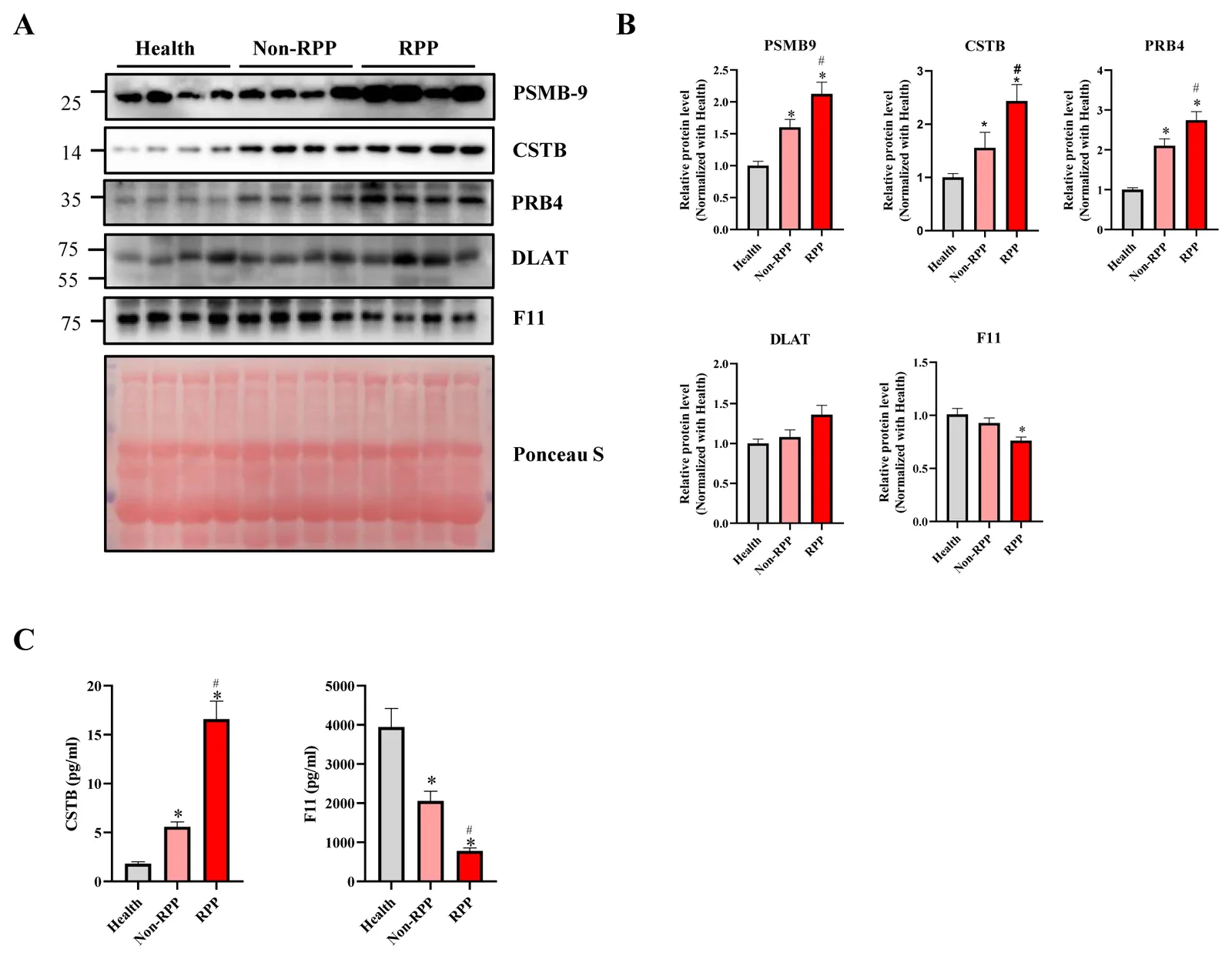
Protein levels of the selected candidates were examined in an independent validation cohort. **(A, B)** Representative Western blots and quantitative analysis showing the expression levels of PSMB9, CSTB, PRB4, DLAT, and F11 in plasma samples from 16 healthy controls, 16 non-RPP patients and 16 RPP patients. **(C)** Concentrations of CSTB and F11 in plasma were measured by ELISA from 16 healthy controls, 30 non-RPP patients and 30 RPP patients. Data are presented as mean ± SD. *p < 0.05 versus the healthy controls, ^#^*p* < 0.05 versus the non-RPP patients.

We validated the expression level of five lung function-related proteins (*p* < 0.05) by WB analysis in 16 RPP patients, 16 non-RPP patients and 16 healthy controls. Consistent with the tendency found in proteomic analysis, the results showed similar alterations in this independent cohort (**Figures 6A, B**). Compared with the non-RPP group, the RPP group exhibited significantly higher protein levels of PSMB9, CSTB and PRB4, whereas the level of F11 was significantly lower. Furthermore, the expression of PSMB9, CSTB and PRB4 showed a stepwise increase from healthy individuals to non-RPP patients and to RPP patients (**Figures 6A, B**). Moreover, ELISA analysis in 30 RPP patients, 30 non-RPP patients and 16 healthy controls also revealed significantly higher expression levels of CSTB and reduced expression of F11 in patients with RPP (**Figure 6C**).

### 3.6 Validation of key proteins in a crystalline silica-induced cell model

**Figure 7:**
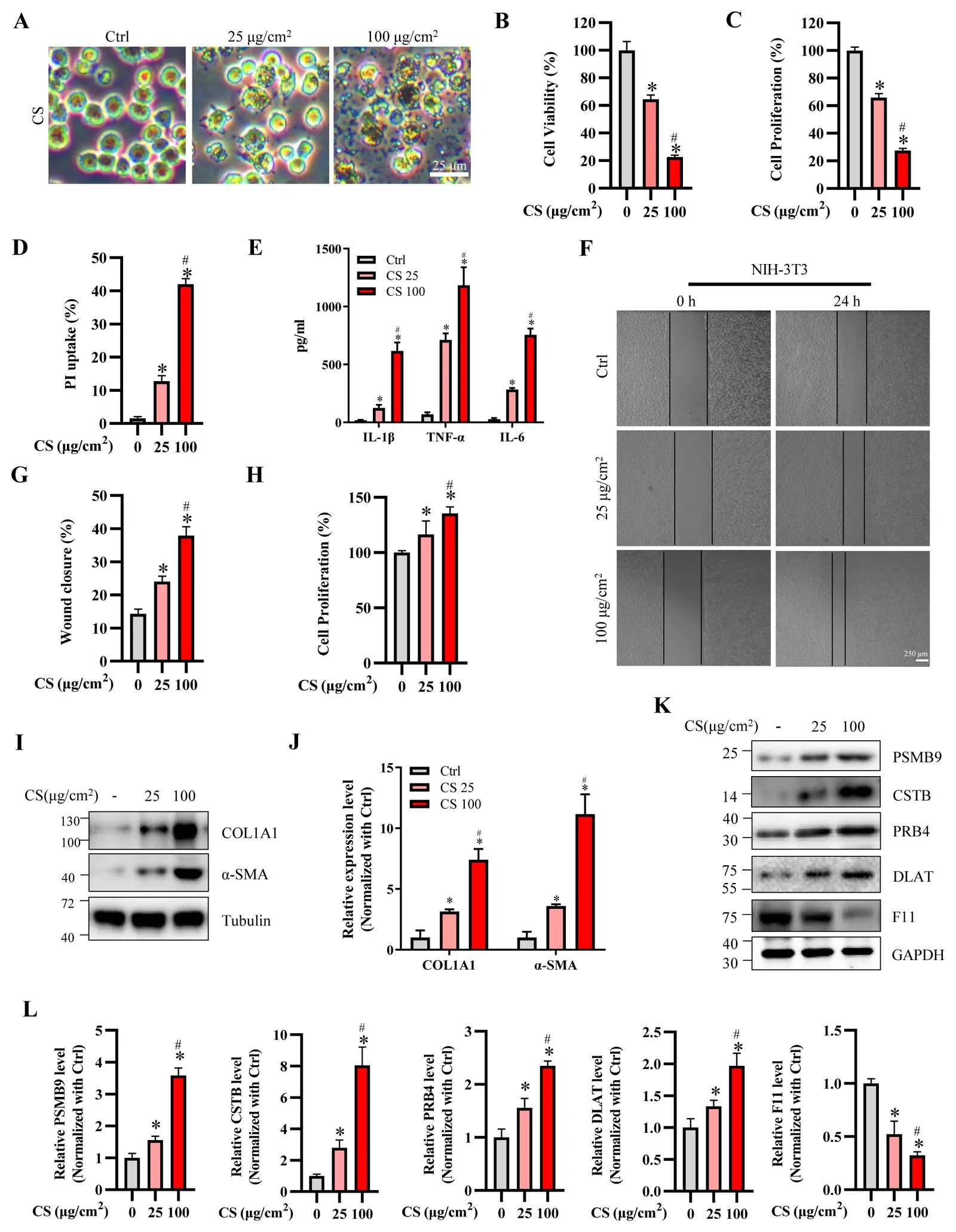
Verification of candidate biomarkers in an *in vitro* RPP-like model. MH-S macrophages were treated with or without crystalline silica **(CS)** at 25 or 100 μg/cm² for 24 h. **(A)** Representative bright-field images showing morphological changes in MH-S cells following CS exposure. Scale bar, 100 μm. Cell viability **(B)**, proliferation **(C)**, and PI uptake **(D)** were assessed using MTT, CCK-8, and flow cytometry assays, respectively. **(E)** Levels of IL-1β, TNF-α, and IL-6 in culture supernatants were measured by ELISA. NIH-3T3 fibroblasts were then incubated with conditioned medium (CM) collected from MH-S cells treated as described above. **(F)** Representative images of wound healing assays at 0 and 24 h after CM treatment. Scale bar, 250 μm. **(G)** Quantitative analysis of wound closure rates. **(H)** Fibroblast proliferation was evaluated by CCK-8 assay following CM exposure. **(I)** Western blot analysis of COL1A1 and α-SMA expression in NIH-3T3 cells after CM treatment, with quantification shown in **(J)**. **(K)** Representative Western blots and **(L)** quantification of PSMB9, CSTB, PRB4, DLAT, and F11 expression in NIH-3T3 cells. Data are presented as mean ± SD from at least three independent experiments. *p < 0.05 versus control group; ^#^*p* < 0.05 versus 25 μg/cm² CS group.

To further investigate the key proteins associated with RPP, we established an RPP-like cell model using MH-S cells exposed to CS. Prior to subsequent biological analyses, we evaluated the cytotoxic effects of a range of CS concentrations (0, 25, 50, 100, and 150 μg/cm²) to determine appropriate exposure conditions (**Supplementary Figure 2**). Increasing CS concentrations resulted in a progressive reduction in MH-S cell viability, whereas exposure to 150 μg/cm² CS caused excessive cell death and was therefore excluded from further experiments. Epidemiological evidence has shown that higher doses of respirable CS are associated with an increased risk of RPP (4, 29). In our experimental system, exposure to 100 μg/cm² CS induced marked cell death in MH-S cells, as evidenced by the abundant cellular debris observed. However, exposure to 25 μg/cm² CS induced only partial cell damage, as evidenced by limited membrane integrity loss (**Figure 7A**; **Supplementary Figure 2A**). The MTT (**Figure 7B**; **Supplementary Figure 2B**) and CCK-8 (**Figure 7C**; **Supplementary Figure 2C**) assays also revealed that exposure to high dose CS significantly inhibited MH-S cell viability and proliferation compared to lower dose. To further assess the impact of CS at varying concentrations on MH-S cells, a PI uptake assay was performed. The results demonstrated a more significant increase in PI uptake at 100 μg/cm² CS, indicating more substantial cell membrane damage (**Figure 7D**; **Supplementary Figure 3**). The cell model was further validated by assessing key pathological features, including elevated levels of pro-inflammatory cytokines, such as IL-1β, TNF-α, and IL-6 (**Figure 7E**). Based on these findings, MH-S cells exposed to 25 μg/cm² and 100 μg/cm² CS were selected to establish *in vitro* models of non-RPP-like and RPP-like, respectively.

In addition to releasing inflammatory factors that directly exacerbate inflammation, AMs contribute to the development of a pro-fibrotic microenvironment by secreting cytokines and chemokines that recruit and activate fibroblasts, thereby driving fibrotic progression (39, 40). NIH-3T3 cells, a well-characterized murine fibroblast cell line, have been extensively utilized as a model system for investigating fibrotic mechanisms in pneumoconiosis research (41–43). To recapitulate the rapid fibrotic process of RPP, NIH-3T3 fibroblasts were stimulated with CM derived from MH-S macrophages. Wound healing and CCK-8 assays revealed that CM from MH-S cells treated with 100 μg/cm² CS significantly enhanced fibroblast migration (**Figures 7F, G**) and increased cell viability (**Figure 7H**). To assess fibroblast activation, the protein levels of collagen type I alpha 1 (COL1A1) and alpha-smooth muscle actin (α-SMA) were measured. Notably, NIH-3T3 cells incubated with CM from the high dose CS-treated group exhibited markedly elevated expression of COL1A1 and α-SMA (**Figures 7I, J**). This cellular model establishes a robust platform for investigating the molecular mechanisms underlying RPP and screening potential therapeutic interventions. Then, we validated the expression levels of PSMB9, CSTB, PRB4, DLAT and F11 by WB analysis. Consistent with our proteomics data, the RPP group (exposed to 100 μg/cm² CS) showed the upregulated protein levels of PSMB9, CSTB, PRB4 and DLAT and the downregulated expression of F11 (**Figures 7K, L**).

## 4 Discussion

RPP represents the most aggressive clinical phenotype of pneumoconiosis characterized by accelerated pulmonary fibrosis, rapid decline in pulmonary function, and poor prognosis (4–6). Although the clinical features of RPP have been increasingly recognized, the systemic molecular alterations associated with this aggressive phenotype remain poorly characterized. In the present study, we integrated DIA-based plasma proteomics and bioinformatic analyses to systematically profile the plasma proteomic landscape associated with RPP. Our findings reveal that RPP is accompanied by coordinated alterations in multiple biological processes at the systemic level, particularly immune-inflammatory activation, metabolic remodeling, and coagulation dysregulation. Notably, the identified proteins do not operate in isolation but form interconnected molecular networks that correlate with accelerated disease progression and declining pulmonary function, suggesting their potential relevance as candidate biomarkers. However, given the exploratory nature of this study, these findings should be interpreted as hypothesis-generating, and the post-hoc power analysis was not performed for the discovery phase; rather, the independent validation cohort provides the primary evidence supporting the robustness of our key observations.

Unlike ordinary pneumoconiosis, which generally progresses slowly over decades, RPP exhibits rapid radiological deterioration and severe functional impairment within a relatively short period (4, 5). Current evidence suggests that excessive crystalline silica exposure induces persistent activation of alveolar macrophages, resulting in intense inflammation, fibroblast activation, and accelerated extracellular matrix deposition (4, 29). Consistent with this concept, our plasma proteomic analysis revealed significant enrichment of pathways involved in macrophage activation, glycolysis/gluconeogenesis, extracellular matrix organization, neutrophil extracellular trap formation, and complement and coagulation cascades. These findings suggest that rapid progression of pneumoconiosis is not driven by a single pathological event but rather by a complex interplay among inflammatory responses, cellular metabolic adaptation, and tissue remodeling. Therefore, the screened proteins should be interpreted as molecular indicators reflecting these coordinated pathological processes rather than isolated biomarkers.

Among the biological processes identified in this study, immune-inflammatory activation emerged as a dominant molecular feature of RPP. Silica particles are known to induce persistent activation of alveolar macrophages, leading to the release of inflammatory cytokines, oxidative mediators, and pro-fibrotic factors that promote fibroblast activation and pulmonary fibrosis (44, 45). Consistent with previous studies, our enrichment analysis identified macrophage-related signaling as one of the major altered pathways in RPP. In particular, macrophage colony-stimulating factor-related signaling was significantly enriched, supporting the concept that enhanced macrophage survival and activation contribute to the sustained inflammatory microenvironment characteristic of RPP (46, 47). Although macrophage colony-stimulating factor expression is generally low in normal lung tissue (48, 49), persistent silica exposure may induce abnormal macrophage activation and amplify inflammatory responses, thereby accelerating fibrotic progression. Several immune-associated proteins identified in our proteomic analysis further support this interpretation. SPP1 is an immune-related glycoprotein secreted by various immune cells (50), and its dysregulation has been observed in silicosis (51, 52). Similarly, ANXA2 is a specific bleomycin target, and bleomycin binding with ANXA2 impedes TFEB-induced autophagic flux, leading to induction of pulmonary fibrosis (53, 54). Together, these findings confirm that the plasma proteomic changes identified in our study are biologically consistent with established mechanisms of silica-induced lung injury and further suggest that persistent immune activation is a key driver of RPP progression. Beyond these pathway-level alterations, the validated proteins PSMB9 and CSTB provide additional insights into the specific molecular components underlying immune dysregulation in RPP. Rather than representing independent inflammatory markers, these proteins likely reflect different aspects of immune remodeling, including immunoproteasome activation (55) and lysosomal homeostasis (56). Their alterations in patient plasma, together with their associations with pulmonary function, suggest that these molecules may contribute to or reflect the persistent inflammatory state that distinguishes RPP from ordinary pneumoconiosis.

In addition to persistent immune activation, our findings indicate that metabolic remodeling represents another important feature associated with the rapid progression of pneumoconiosis. Cellular metabolism is increasingly recognized as a critical regulator of chronic inflammation and fibrotic remodeling, as activated immune cells and fibroblasts undergo metabolic adaptation to support sustained inflammatory signaling, proliferation, and extracellular matrix production (57). Consistent with this concept, our proteomic analysis identified significant enrichment of glycolysis/gluconeogenesis-related pathways in RPP, suggesting that metabolic reprogramming may participate in the transition from silica-induced inflammation to progressive fibrosis. Among the validated proteins, DLAT represents a potential molecular link between altered metabolism and fibrotic progression. DLAT is an essential component of the pyruvate dehydrogenase complex, which controls the conversion of pyruvate into acetyl-CoA and determines the metabolic flux between glycolysis and mitochondrial oxidative metabolism (58). Previous studies have demonstrated that metabolic abnormalities, including enhanced glycolytic activity and mitochondrial dysfunction, contribute to silica-induced pulmonary fibrosis by promoting fibroblast activation and extracellular matrix accumulation (59). In addition, altered DLAT expression has been associated with metabolic adaptation during fibrotic processes (60, 61). These findings suggest that DLAT may reflect metabolic alterations accompanying disease progression rather than representing a nonspecific consequence of lung injury.

Another notable finding of the present study was the enrichment of complement and coagulation-related pathways, suggesting that alterations in vascular and coagulation homeostasis may also contribute to RPP progression. Although coagulation pathways have traditionally been considered primarily responsible for hemostasis, increasing evidence indicates that coagulation factors function as important regulators of inflammation, endothelial injury, immune cell recruitment, and tissue remodeling (62, 63). Dysregulated coagulation signaling has been implicated in several chronic pulmonary diseases (64), including idiopathic pulmonary fibrosis, where coagulation activation interacts with inflammatory pathways to promote fibroproliferative responses (65). Among the identified biomarkers, F11 was significantly altered in patients with RPP and showed a strong association with pulmonary function. F11 is a key procoagulant hemostatic factor and has been increasingly recognized as a mediator linking coagulation and inflammation (66). Although the specific role of F11 in silica-induced pulmonary fibrosis remains unclear, its dysregulation in RPP suggests that altered coagulation homeostasis may represent an additional layer of pathological regulation during rapid disease progression. Taken together, the identification of DLAT and F11 extends the molecular understanding of RPP beyond inflammation alone. While immune-related proteins reflect persistent inflammatory activation, DLAT and F11 indicate that metabolic adaptation and coagulation imbalance are also involved in the disease process. These findings suggest that immune activation, metabolic remodeling, and coagulation dysregulation may represent interconnected systemic alterations associated with the clinical trajectory of RPP.

Thus, these findings delineate a systemic molecular landscape of RPP, in which PSMB9 and CSTB reflect immune-inflammatory remodeling, DLAT represents metabolic adaptation, F11 indicates alterations in coagulation homeostasis, and PRB4 may be involved in cell growth-related processes (67). These complementary signatures suggest that RPP is characterized by coordinated dysregulation across multiple biological systems rather than by a single dominant pathway. This integrated view also explains why multiple analytical strategies were required for candidate prioritization, as differential expression analysis captures disease-associated alterations, protein-protein interaction analysis highlights functional connectivity, and machine learning ranks proteins by discriminatory ability. Together, these complementary approaches enabled the identification of candidate biomarkers that are not only statistically associated with RPP but also functionally interconnected.

Several limitations should be acknowledged. First, the discovery cohort comprised only 28 patients (15 RPP vs. 13 non-RPP), which limits statistical power and generalizability. To reduce the risk of false-positive findings, we selected patients with maximally contrasting clinical phenotypes and applied a multilayered screening strategy incorporating LASSO regression with 100 bootstrap resampling iterations, PPI network analysis, and machine-learning algorithms. The prioritized candidate proteins were subsequently validated in an independent cohort using WB and ELISA and further verified in cell models. Nevertheless, the relatively limited size and single-center nature of the validation cohort restrict the robustness and generalizability of these findings. Accordingly, our results should be considered hypothesis-generating, providing a prioritized set of candidate biomarkers that require further validation in larger, multicenter cohorts using standardized quantitative assays. Second, COPD represents a common comorbidity in pneumoconiosis and may introduce confounding. Although the prevalence of COPD was comparable between groups and subgroup analyses excluding COPD patients revealed consistent protein expression patterns (**Supplementary Figure 4**), the possibility of residual confounding by COPD cannot be excluded. Furthermore, the absence of other pulmonary disease controls (e.g., COPD, asthma) limits our ability to assess the specificity of the identified signatures to RPP versus other respiratory conditions. Of note, healthy controls were included only in the validation cohort, where a progressive stepwise alteration from healthy controls to non-RPP patients and further to RPP patients was observed. Future studies incorporating comprehensive COPD phenotyping, disease control groups and larger healthy control cohorts are warranted to further validate our findings. Third, the distribution of pneumoconiosis stages was uneven between groups, with no stage III patients in the non-RPP cohort. A subgroup analysis comparing Class 1 RPP patients (progression without PMF) to non-RPP controls yielded largely consistent protein expression patterns (**Supplementary Figure 4**), suggesting that these signals are not solely attributable to PMF-related severity. Nonetheless, some identified proteins may still reflect advanced disease stage or PMF burden, and future longitudinal studies with serial sampling will be necessary to distinguish progression-specific changes. Fourth, the pneumoconiosis subtype distribution was also imbalanced between groups. Although this difference was not statistically significant, we acknowledge that the numerical imbalance may introduce confounding. Nevertheless, pathological evidence indicates that crystalline silica drives rapid fibrosis in both silicosis and coal workers’ pneumoconiosis (4), and our *in vitro* model further supports that the observed signals reflect RPP characters rather than dust subtype differences. Thus, future studies with balanced subtype representation are needed. Fifth, it should also be noted that the *in vitro* model established in this study was primarily designed as a biological validation platform rather than a mechanistic model. Although the selected CS concentrations generated biologically distinct inflammatory–fibrotic conditions for validating candidate proteins identified by plasma proteomics, intermediate CS concentrations were not systematically evaluated. Given that no standardized *in vitro* model currently exists for recapitulating the pathological progression of RPP, this system should be regarded as an experimental approximation. Future studies employing more physiologically relevant culture systems and a broader range of CS concentrations will help characterize dose-dependent cellular responses, further optimize the *in vitro* model, and better recapitulate the spectrum of silica-induced inflammatory and fibrotic alterations. In addition, genetic or pharmacological manipulation of individual candidate proteins will be required to further elucidate their mechanistic roles in silica-induced inflammation and fibrosis. Finally, the absence of paired plasma-lung tissue or plasma- bronchoalveolar lavage fluid samples precludes any empirical assessment of the correlation between plasma protein abundance and local pulmonary expression. Observed plasma changes could alternatively arise from systemic inflammation, comorbidities, protein clearance, or extrapulmonary sources. Nevertheless, several of our identified DEPs, including SPP1, S100A8, ADIPOQ, and CAPN2, have been independently reported to be dysregulated during pulmonary injury and fibrosis in relevant experimental models (68–71), lending indirect biological plausibility to our findings. Taken together, these limitations underscore that this study should be interpreted as a hypothesis-generating plasma biomarker discovery effort, with all candidate proteins requiring validation in larger, well-characterized cohorts before any definitive conclusions can be drawn.

In conclusion, this hypothesis-generating study provides a foundational plasma proteomic characterization of RPP and nominates candidate biomarkers for future investigation. The observed coordinated alterations across immune, metabolic, and coagulation pathways highlight the systemic complexity of this aggressive phenotype, though these findings remain correlative and require causal validation. The candidate proteins identified, particularly PSMB9, CSTB, PRB4, DLAT, and F11, represent promising targets for independent validation in larger, well-characterized cohorts.

## Data Availability

The datasets presented in this study can be found in online repositories. The names of the repository/repositories and accession number(s) can be found below: PXD063647 (ProteomeXchange; https://proteomecentral.proteomexchange.org/cgi/GetDataset?ID=PXD063647).

## Supplementary material

Supplementary Figure 1 The quality evaluation of mass spectrometry data from 15 RPP and 13 non-RPP patients. **(A)** Density distribution curve of identified proteins. **(B)** Number of peptides length. **(C)** The distribution of proteins among different molecular weight. **(D)** The distribution of protein intensity values among different samples.

Supplementary Figure 2 Selection of crystalline silica concentrations for establishing the *in vitro* validation model. **(A)** Representative bright-field images of MH-S macrophages following exposure to crystalline silica (CS) at indicated concentrations (0, 25, 50, 100, and 150 μg/cm²) for 24 h. Scale bar, 25 μm. **(B)** Cell viability determined by MTT assay. **(C)** Cell viability determined by CCK-8 assay. Data are presented as mean ± SD from at least three independent experiments. **p* < 0.05 versus control group.

Supplementary Figure 3 Flow cytometry analysis of MH-S cells after 24 h exposure to CS (25 or 100 μg/cm²) using PI staining.

Supplementary Figure 4 Validation of candidate biomarkers in subgroup analyses excluding COPD and PMF-related bias. Protein expression levels of PSMB9, F11, CPPED1, CSTB, DLAT, and PRB4 were compared between non-RPP patients and the overall RPP cohort **(A)**, as well as between non-RPP patients and RPP subgroups after excluding COPD **(B)** or PMF (RPP-ILO subgroup, **C**). Data are presented as mean ± SEM.
